# RNA interactomics: recent advances and remaining challenges

**DOI:** 10.12688/f1000research.16146.1

**Published:** 2018-11-20

**Authors:** Brigitte Schönberger, Christoph Schaal, Richard Schäfer, Björn Voß

**Affiliations:** 1 Institute of Biochemical Engineering, Computational Biology Group, University of Stuttgart, Stuttgart, 70569, Germany

**Keywords:** RNA, RNA-RNA interactions, Regulation

## Abstract

Tight regulation of cellular processes is key to the development of complex organisms but also vital for simpler ones. During evolution, different regulatory systems have emerged, among them RNA-based regulation that is carried out mainly by intramolecular and intermolecular RNA–RNA interactions. However, methods for the transcriptome-wide detection of these interactions were long unavailable. Recently, three publications described high-throughput methods to directly detect RNA duplexes in living cells. This promises to enable in-depth studies of RNA-based regulation and will narrow the gaps in our understanding of RNA structure and function. In this review, we highlight the benefits of these methods and their commonalities and differences and, in particular, point to methodological shortcomings that hamper their wider application. We conclude by presenting ideas for how to overcome these problems and commenting on the prospects we see in this area of research.

## Introduction

RNA-based regulation is an important regulatory layer in all domains of life, carried out by a multitude of non-coding RNAs (ncRNAs). They interact with proteins, RNA, and DNA and thereby interconnect different regulatory systems. The elucidation of the underlying networks is thus of central importance for a holistic understanding of cellular regulation. Computational prediction based on sequence alone turns out to be unsatisfactory, and, with the availability of second- and third-generation sequencing technologies, data-based inference of regulatory networks involving ncRNAs is the more promising approach. RNA–protein interactions are more or less routinely studied using crosslinking immunoprecipitation-sequencing (CLIP-seq) ^1^, RNA immunoprecipitation-sequencing (RIP-seq) ^2^, mapping RNA interactome *in vivo* (MARIO) ^3^, or related methods, which rely on ultraviolet-induced crosslinking or affinity of proteins to RNA; specific complexes are then enriched by immunoprecipitating the RNA-binding protein of interest, followed by sequencing of the RNA content using RNA sequencing (RNA-seq) (see recent reviews ^4, 5^).

A large fraction of ncRNAs carry out their function through complementary base pairing to other RNAs, often mRNAs. This may involve proteins, as in the case of the RISC complex for microRNA (miRNA)- or small interfering RNA (siRNA)-mediated post-transcriptional gene silencing, but the ncRNA is the main factor that specifies the target RNAs via base complementarity. Thus, unbiased methods for the transcriptome-wide discovery of direct RNA–RNA interactions are needed to decipher RNA regulatory networks. Approaches developed in 2016, described as Direct Duplex Detection (DDD) methods ^6^, represent an important first step toward this goal. In the following, we will discuss their pros and cons and present our view on what future improvements may look like and what their impact might be.

## State of the art

RNA–RNA interactions pose two basic problems when analyzing them. First, they are commonly disrupted during the process of RNA extraction and cannot be reconstituted to their native state *in vitro*. Second, there is no technology available that can sequence interacting RNA strands and maintain their relationship. The first approach to tackle these problems (in a rather coarse way) was described by Ramani *et al*. ^7^. They presented RNA proximity ligation (RPL), in which interacting RNA strands are ligated to each other in a standard ligation reaction using RNase inhibitor-treated crude cell extracts followed by RNA-seq. The resulting sequencing reads contained chimeric reads but to a rather low fraction (0.28%). Nevertheless, the proximity ligation approach presents a solution to the abovementioned second problem because it allows one to trace back RNA–RNA interactions in data sets obtained by next-generation sequencing technologies.

The remaining problem of losing RNA–RNA interactions due to the harsh RNA extraction protocols is as long-standing as its solution: direct RNA crosslinking via psoralen derivatives. Following irradiation, psoralens covalently and reversibly connect two pairing stretches of RNA. Thus, duplexes are maintained during RNA extraction, which enables their enrichment, for example, by nucleases that digest single-stranded RNAs. The psoralen-mediated *in vivo* crosslinking of RNAs has been known since the 1970s and successfully used to interrogate the structure of ribosomal RNAs, interactions between small nuclear RNAs, and several other RNA structures and interactions ^8– 20^.

**Figure 1.**
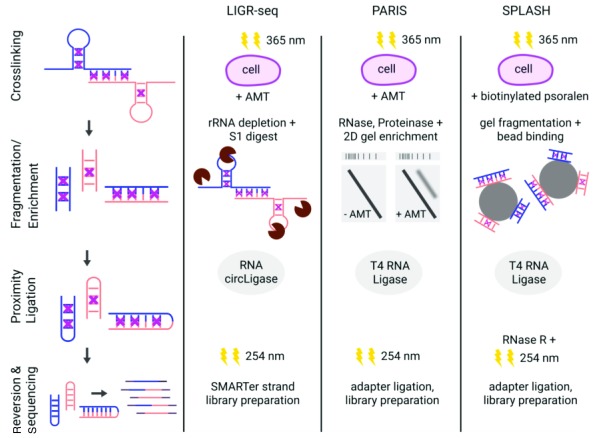
Schematic comparison of Direct Duplex Detection methods. Overview of the protocols for LIGR-seq ^21^, PARIS ^22^, and SPLASH ^23^. The first column shows the principal steps of the three experimental procedures: crosslinking (violet crosses) was conducted *in vivo* by 365 nm irradiation and 4′-aminomethyltrioxsalen (AMT) or biotinylated psoralen treatment. Fragmentation was performed enzymatically (LIGR-seq and PARIS) or chemically (SPLASH). Crosslinked RNAs were additionally enriched either by size separation using two-dimensional (2D) gel electrophoresis (crosslinked RNAs above the main diagonal were eluted; PARIS) or by biotin-streptavidin binding to magnetic beads (SPLASH). Proximity ligation was carried out using different ligases. Crosslinks were reverted by 254 nm irradiation. For sequencing, different library preparation strategies were performed. Colors of RNA strands (blue and orange) indicate different RNA molecules. LIGR-seq, ligation of interacting RNA followed by high-throughput sequencing; PARIS, psoralen analysis of RNA interactions and structures; SPLASH, sequencing of psoralen-crosslinked, ligated, and selected hybrids.

The combination of the two aforementioned strategies is the hallmark of the currently available DDD methods, namely ligation of interacting RNA followed by high-throughput sequencing (LIGR-seq) ^21^, psoralen analysis of RNA interactions and structures (PARIS) ^22^, and sequencing of psoralen-crosslinked, ligated, and selected hybrids (SPLASH) ^23^. Although they differ in several steps of their experimental protocols, they all share the same principal design, which is *in vivo* RNA crosslinking followed by RNA extraction, enrichment of crosslinked RNAs, proximity ligation, and sequencing via RNA-seq. A comparison of the individual methods is shown in Figure 1.

A common bias that occurs when psoralen is used to crosslink double-stranded RNA (dsRNA) is its preference to intercalate into adjacent opposite pyrimidine bases, preferably uracil ^24^, meaning that interactions in GC-rich regions may be under-represented. Nevertheless, the chance that uracil residues are neighboring another base pair is 25%, assuming a uniform distribution of all possible pairs of bases.

The major problem of all of the abovementioned methods is that the proximity ligation step is highly inefficient and that it is not possible to perform an RNA-seq library preparation that enriches for or specifically targets the ligated RNAs. Furthermore, the proximity ligation step does not exclusively ligate interacting RNA strands but also all kinds of single-stranded RNAs in a random fashion. As a result, the yield of RNA–RNA interaction informative reads is very low, as shown in Table 1.

**Table 1.** Comparison of read statistics.

	LIGR-seq	PARIS	SPLASH
Total number of sequencing reads	171,239,817	99,698,824	189,340,955
Chimeric reads	6,614,251 (~3.9%)	2,077,743 (~2%)	1,038,801 (~0.5%)
RNA–RNA interactions	1,029	232,031a	4,026

Data derived from psoralen-treated human cell line samples (LIGR-seq and PARIS: total RNA isolated from HEK293T human embryonic kidney cells; SPLASH: total RNA from GM12892 human lymphoblastoid cells); all replicates included. Values are taken from the supplementary information of the respective publication (LIGR-seq ^21^, PARIS ^22^, and SPLASH ^23^). In the case of LIGR-seq, the number of chimeric reads was determined by the corresponding analysis pipeline Aligater ^21^. ^a ^So-called Duplex Groups, representing gapped reads with interacting RNA sites. LIGR-seq, ligation of interacting RNA followed by high-throughput sequencing; PARIS, psoralen analysis of RNA interactions and structures; SPLASH, sequencing of psoralen-crosslinked, ligated, and selected hybrids.

Nevertheless, the presented approaches provided interesting and deep insights into the RNA–RNA interaction networks of the studied organisms and the respective growth conditions. With the help of these DDD methods, already experimentally verified RNA structures could be accurately recapitulated; in addition, novel RNA structures as well as new RNA–RNA interactions were revealed. For instance, LIGR-seq identified novel interactions between small nucleolar RNAs (snoRNAs) and mRNAs, including the snoRNA SNORD83B that controls the steady-state levels of its target mRNAs ^21^. Data from PARIS support the results of Lin *et al*. ^25^, who discovered that the interactions within the long ncRNA NEAT1 have an important architectural function in paraspeckle formation and thus impact the regulation of gene expression. In addition, PARIS could detect inter-repeat duplexes in the long ncRNA XIST, which is essential for X chromosome silencing ^22^. Furthermore, the use of SPLASH has uncovered the RNA–RNA interactome of influenza A viruses required for virus growth ^26^; this might facilitate the prediction of the emergence of new pandemic influenza strains.

## Conclusion and perspective

Even based on the highly inefficient proximity ligation, the currently available DDD methods have been able to capture and analyze crucial RNA–RNA interactions in a transcriptome-wide fashion and *in vivo.* Nevertheless, there is still room for improvement. A stricter enrichment of crosslinked RNAs by combining enzymatic digestions (LIGR-seq) with two-dimensional gel-electrophoretic separation (PARIS) or biotinylated-psoralen selection (SPLASH) or both could eliminate the undesired fortuitous ligation of single-stranded or non-crosslinked RNA duplexes.

The efficiency of the proximity ligation could be enhanced with non-complementary overhangs. These may be either left over by a limited nuclease digestion or introduced synthetically before ligation, for example, by polyA tailing or by using different terminal transferases. Such duplex overhangs are more likely to be ligated and could lead to improved yields of interaction-informative sequencing reads.

The most straightforward approach to turn RNA duplexes into sequencing templates would be a direct dsRNA ligation method. So far, only one report has proposed using RNA and DNA ligases for the enzymatic joining of two or more dsRNA molecules ^27^. However, to the best of our knowledge, the results have never been reproduced, nor was the method used in further studies. In addition, future discoveries might provide engineered DNA ligases with substrate promiscuity to increase the dsRNA ligation efficiency. Eventually, a direct ligation of dsRNA adapters would pave the way for novel and hopefully more-efficient DDD protocols.

Likewise, the bioinformatics pipelines for basic data analysis and statistical inference of interactions need to be improved. Currently, none of the existing software packages considers the actual complementarity of the putative interaction partners but relies only on statistical measures. Furthermore, RNA–RNA interactions are driven by base-pair formation, for which thermodynamic parameters and kinetic models exist. Integrating these aspects into the analyses may additionally improve the reliability and robustness of data analysis.

In conclusion, the currently available DDD methods pave the way to a deeper understanding of the so-far little-studied RNA–RNA interactome. Crucial for their future acceptance and success will be to increase the yield of informative reads, which would allow RNA regulatory networks to be deciphered in greater depth. Results from such studies could have a great impact on many research areas. For example, a lot of human diseases are associated with ncRNAs (for example, Alzheimer’s disease ^28^, schizophrenia ^29^, and various cancer types ^30^ such as leukemia ^31^, breast cancer ^32^, or lung cancer ^33^). Thus, there is and will be a wide application range for first- and next-generation DDD methods that have the potential to provide important contributions in areas of basic and applied sciences.
